# Upregulation of Caveolin-1 in breast cancer and its potential value in distinguishing TNBC from non-TNBC: an immunohistochemical study

**DOI:** 10.3389/fonc.2026.1797743

**Published:** 2026-04-15

**Authors:** Chao-Qun Wang, Jun-Kang Shao, Yan Wang, Shi-Ping Lu, Zheng-Guo Xu, Chao-Hui Hu, Bi-Fei Huang

**Affiliations:** 1 Department of Pathology, Affiliated Dongyang Hospital of Wenzhou Medical University, Dongyang, Zhejiang, China; 2 Department of Medical Oncology, Affiliated Dongyang Hospital of Wenzhou Medical University, Dongyang, Zhejiang, China

**Keywords:** breast cancer, CAV-1, Caveolin-1, immunohistochemistry, prognosis

## Abstract

Currently, there are few studies exploring the expression of Caveolin-1 (CAV-1) in breast cancer tissues and its clinicopathological significance using immunohistochemistry (IHC) technology. This study detected the expression of CAV-1 in 300 cases of invasive breast cancer paraffin tissues through IHC, and analyzed the relationship between CAV-1 expression and the clinicopathological characteristics and prognosis of breast cancer patients. Our results showed that the positivity rate of CAV-1 in breast cancer tissues was 10.0% (30/300), significantly higher than that in normal breast tissues (0.0%, 0/58) (*P* = 0.024). CAV-1 expression was higher in breast cancer with larger tumors, higher tumor grade, ER negative, PR negative, HER2 negative, higher Ki-67 index, and triple-negative breast cancer (TNBC) molecular subtype (all *P* < 0.05); however, the positivity rate of CAV-1 in breast cancer without axillary lymph node metastasis (13.6%, 21/154) was significantly higher than that in patients with axillary lymph node metastasis (4.9%, 7/143) (*P* = 0.010). In multiple logistic regression analysis, the independent predictor of CAV-1 expression in breast cancer was TNBC status (odds ratio = 32.099; 95% confidence interval: 9.341–110.302; *P* < 0.001). We further analyzed the value of CAV-1 in distinguishing between TNBC and non-TNBC, the results showed that the sensitivity, specificity, positive predictive value (PPV), and negative predictive value (NPV) of CAV-1 positive prediction for TNBC were 32.9%, 98.6%, 90.0%, and 79.5%, respectively. Regardless of all breast cancer or TNBC patients, no clear relationship was observed between patient prognosis and CAV-1 expression. We suggest that CAV-1 expression is significantly upregulated in TNBC, with potential clinical value for distinguishing between TNBC and non-TNBC.

## Introduction

Female breast cancer is the most commonly diagnosed cancer worldwide and is the leading cause of cancer-related death among women, with significant heterogeneity in terms of prognosis and treatment response (1, 2). Caveolin-1 (CAV-1) is a scaffolding protein and the primary structural component of caveolae, which are flask-shaped invaginations of the plasma membrane. It interacts with various membrane proteins and signaling molecules to regulate cellular processes, including signal transduction, cholesterol homeostasis, cell proliferation, and tumorigenesis (3–6). Consistently, previous studies have implicated CAV-1 in the pathogenesis and progression of breast cancer (7–9). However, CAV-1 expression in breast cancer tissues and its clinicopathological significance have not been extensively studied using the most commonly used immunohistochemistry (IHC) technology in clinical pathology work. This type of study found only a small number of records, one of which applied IHC to detect CAV-1 expression in 80 cases of breast cancer tissues after Neoadjuvant chemotherapy (NACT). The results showed that high levels of CAV1 were significantly associated with survival in these patients (10). Another study explored immunohistochemical and molecular genetic characteristics of 37 invasive apocrine carcinomas of the breast using technologies such as immunohistochemistry (IHC), fluorescent *in situ* hybridization (FISH), and next-generation sequencing (NGS) assays. In the IHC results, infrequent (~10%) expression of CAV-1 was shown (11). The number of cases included in these studies is relatively small, or only involves a specific subtype of breast cancer, therefore failing to elucidate the role and clinical value of CAV-1 in breast cancer.

This study aimed to fill this gap by investigating the expression of CAV-1 in 300 cases of invasive breast cancer paraffin tissues through IHC analysis. The relationship between CAV-1 expression and the clinicopathological characteristics and prognosis of breast cancer patients was also explored. Understanding the role of CAV-1 in breast cancer is essential for the development of novel therapeutic strategies and personalized medicine approaches. By analyzing CAV-1 expression in a large cohort of breast cancer patients and correlating it with clinical outcomes, this study seeks to provide valuable insights into the clinical significance of CAV-1 in breast cancer.

## Materials and methods

### Patients and tissue samples

Tissue samples of breast cancer were collected from 300 Chinese Han women who had undergone surgery for breast cancer at the Affiliated Dongyang Hospital of Wenzhou Medical University (Dongyang, Zhejiang, China) from 2007 to 2019. Contains 287 cases of invasive ductal carcinoma, 5 cases of medullary carcinoma, 6 cases of metaplastic carcinoma and 2 cases of invasive micropapillary carcinoma. Fifty-eight samples of adjacent normal breast tissue were also obtained following surgical resection. The adjacent normal breast tissues were defined as “normal” only after being confirmed by two pathologists and were located at least 3cm from the tumor margin. These tissues were verified to consist of benign breast lobules and ducts, with no evidence of malignancy or pre-malignant lesions. Inclusion criteria: a patient’s surgical specimen was diagnosed with invasive breast cancer by pathological diagnosis, and the participants or the participants’ legal guardians/next of kin agreed to participate in the scientific research; exclusion criteria: patients who underwent any antitumor treatment, such as targeted therapy, chemotherapy, immunotherapy, or radiotherapy, prior to surgery were excluded from this study. Breast cancer patients were aged between 24 and 84 years, with a median age of 51 years. A pathohistological diagnosis was made according to breast tumor classification criteria of the World Health Organization (12, 13). Histological grading was based on the Scarff-Bloom-Richardson system (14). According to estrogen receptor (ER), progesterone receptor (PR) and human epidermal growth factor receptor 2 (HER2) status, tissue samples were classified into triple-negative breast cancer (TNBC) (ER^–^, PR^–^, HER2^–^) or non-TNBC subtype (15–18). The characteristics and grouping information of breast cancer patients can be found in **Table 1**. However, two cases with ER−, PR−, and equivocal HER2 2+ status did not undergo fluorescence *in situ* hybridization (FISH) were not classified into TNBC or non-TNBC groups. Instead, these two cases were included in the HER2-equivocal subgroup presented in **Table 1**, and HER2 status was incorporated as a variable in the logistic regression analysis. Additionally, information on lymph node metastases was absent for 3 cases, ki-67 index was missing for 5 cases, and tumor stage was missing for 2 cases. Follow-up information was available for 278 patients, with a median follow-up period of 60 months (range, 4–60 months). The remaining 22 patients were lost to follow-up due to loss of contact by telephone and absence of postoperative treatment at our hospital. All of the study methodology satisfied the relevant guidelines and regulations issued by the Affiliated Dongyang Hospital of Wenzhou Medical University.

**Table 1:** Association of CAV-1 expression with clinicopathological parameters in breast cancer patients.

Variables	No. of patients	CAV-1 positive, n (%)	*P*-value
Age (years)			
≤35	14	3 (21.4%)	0.111*
36–55	177	13 (7.3%)	
>55	109	14 (12.8%)	
Tumor size (cm)			
≤2	143	9 (6.3%)	**0.041**
>2	157	21 (13.4%)	
Lymph node metastases			
No	154	21 (13.6%)	**0.010**
Yes	143	7 (4.9%)	
Tumor grade			
I	13	0 (0.0%)	**<0.001**
II	183	8 (4.4%)	
III	104	22 (21.2%)	
Tumor stage			
I	87	8 (9.2%)	0.453
II	146	17 (11.6%)	
III–IV	65	4 (6.2%)	
Estrogen receptor			
Negative	134	28 (20.9%)	**<0.001**
Positive	166	2 (1.2%)	
Progesterone receptor			
Negative	163	29 (17.8%)	**<0.001**
Positive	137	1 (0.7%)	
HER2 expression			
Negative (0–1^+^)	144	27 (18.8%)	**<0.001**
Equivocal (2^+^)	87	3 (3.4%)	
Positive (3^+^)	69	0 (0.0%)	
Ki-67			
<14%	126	2 (1.6%)	**<0.001**
≥14%	169	28 (16.6%)	
Molecular classification			
non-TNBC	216	3 (1.4%)	**<0.001**
TNBC	82	27 (32.9%)	
Lymphvascular invasion			
No	127	16 (12.6%)	0.093
Yes	29	0 (0.0%)	

*Pearson’s chi-square test was used for the comparison of the CAV-1 positive expression rate among different groups. A bold value of P<0.05 indicates statistical significance.

### Tissue array preparation and IHC analysis

Tissue Array Preparation: We followed the methods described by Wang et al., 2020 (19). To summarize, the Quick-Ray^®^ UT-06 tissue microarray system and the Quick-Ray premade recipient block (UB-06) wax model, both produced by Unitma Co., Ltd. in Seoul, Korea, were utilized for the preparation of tissue specimens measuring 1 mm in diameter. Two specific locations were chosen from each sample of breast cancer tissue for sampling purposes. IHC Analysis: The Envision System (Dako, Glostrup, Denmark) was used for IHC staining of paraffin-embedded tissue sections, following the method previously described (16, 17). Primary antibodies used in the experiment included the anti-CAV-1 rabbit monoclonal antibody (clone E249; diluted to a concentration of 1:500; obtained from Abcam, Cambridge, England). For the secondary antibody, Dako’s HRP rabbit/mouse universal antibody (from Dako, Glostrup, Denmark) was employed. In the negative control, the vehicle was initially incubated, followed by the secondary antibody, without any primary antibody. As positive control, we used the internal control in the breast tissue, to check for CAV-1 staining in the interstitial fibers and vascular endothelial cells.

### Assessment of staining

CAV-1 staining is mainly located on the cell membrane, with some staining visible in the cytoplasm. In this study, immunostaining was considered positive when ≥1% of breast cancer cells or normal glandular epithelial cells exhibited intact or incomplete membranous staining, whereas cytoplasmic staining was excluded from the evaluation (17, 20). The interpretation of staining results is independently completed by two pathologists. If the results from the two pathologists were not consistent, a third pathologist would evaluate and determine the final score.

### Patient follow-up

Patients were followed-up using previously described methods (18, 19). In summary, each patient was monitored post-surgery through phone calls and every 6 months at hospital appointments; follow-up would stop if the patient passed away. Breast cancer recurrence was diagnosed through clinical imaging or pathological histology. Relapse-free survival (RFS) was determined as the duration between surgery and relapse or metastasis, distant metastasis-free survival (DMFS) was the span between surgery and metastasis, and overall survival (OS) was the time between surgery and death (excluding deaths unrelated to the tumor).

### Statistical analysis

Statistical analysis was carried out using SPSS software version 19.0 (SPSS Inc, Chicago, IL, USA). Differences in CAV-1 expression among groups were compared using a Pearson’s chi-square test for categorical variables. Multivariate logistic regression analysis (forward stepwise (likelihood ratio) method) was utilized to identify independent correlation factors of CAV-1 expression. The predictive ability of CAV-1 for TNBC was assessed by calculating its sensitivity, specificity, positive predictive value (PPV), and negative predictive value (NPV). RFS, DMFS and OS rates were determined using the Kaplan-Meier method and compared with log-rank testing. Multivariate analysis using the Cox proportional hazard model was performed to investigate independent factors prognostic of RFS, DMFS and OS. To assess the interobserver reliability of CAV-1 immunostaining interpretation, the level of agreement was determined by calculating Cohen’s kappa coefficient (κ), with a value of >0.75 considered to indicate excellent agreement. A significance level of *P* < 0.05 was used for statistical analysis.

## Results

### CAV-1 expression in breast tissues

An interobserver agreement analysis was first performed on the breast cancer cohort. The assessment between the two pathologists for CAV-1 immunostaining evaluation revealed a Cohen’s kappa coefficient (κ) of 0.963 (standard error, 0.026; *P* < 0.001).

**Figure 1:**
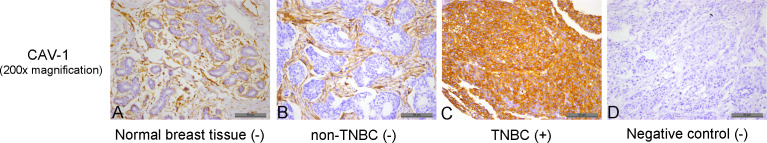
Immunochemical analysis of CAV-1 expression in breast tissues. **(A)** Normal breast tissue, negative CAV-1 expression in normal glandular epithelial cells. **(B)** non-TNBC, negative CAV-1 expression in cancer cells. **(C)** TNBC, positive CAV-1 expression in cancer cells. **(D)** Negative control, all cells in the breast cancer tissue, including cancer cells and stromal cells, show negative expression of CAV-1.

Among 300 cases of breast cancer tissues, 30 were CAV-1 positive (10.0%, 30/300), while the CAV-1 positivity rate in 58 cases of normal breast tissues was 0.0% (0/58) (**Table 2**). The CAV-1 positivity rate in breast cancer tissues was significantly higher than in normal breast tissues (*P* = 0.024) (**Figure 1**).

**Table 2:** CAV-1 expression in breast tissue specimens.

		CAV-1 expression	
Tissue samples	No.	Negative, n (%)	Positive, n (%)
Noncancerous	58	58 (100.0%)	0 (0.0%)
Cancerous	300	270 (90.0%)	30 (10.0%)*

*P < 0.05 vs normal breast tissue.

Furthermore, the CAV-1 positivity rates across different histological subtypes of breast cancer were as follows: among 287 cases of invasive ductal carcinoma, 25 (8.7%) were positive for CAV-1; among 5 cases of medullary carcinoma, 1 (20.0%) was positive; among 6 cases of metaplastic carcinoma, 4 (66.7%) were positive; and among 2 cases of invasive micropapillary carcinoma, none (0.0%) exhibited CAV-1 positivity.

### Relationship between CAV-1 expression and clinicopathological features of breast cancer

As shown in **Table 1**, the positivity rate of CAV-1 in breast cancer patients with larger tumors (>2cm) was 13.4% (21/157), significantly higher than patients with smaller tumors (≤2cm) (6.3%, 9/143) (*P* = 0.041). The positivity rate of CAV-1 in grade III breast cancer patients (21.2%, 22/104) was significantly higher than in grade II (4.4%, 8/183) and grade I (0.0%, 0/13) patients (*P* < 0.001). Furthermore, the positivity rates of CAV-1 in ER negative, PR negative, and higher Ki-67 index (≥14%) breast cancer patients were 20.9% (28/134), 17.8% (29/163), and 16.6% (28/169), respectively, significantly higher than in ER positive (1.2%, 2/166), PR positive (0.7%, 1/137), and low Ki-67 index (<14%) (1.6%, 2/126) patients (all *P* < 0.001). Additionally, the positivity rate of CAV-1 in HER2 negative (0–1+) breast cancer patients (18.8%, 27/144) was significantly higher than in HER2 equivocal (2+) (3.4%, 3/87) and HER2 positive (3+) (0.0%, 0/69) patients (*P* < 0.001). Finally, the positivity rate of CAV-1 in TNBC was 32.9% (27/82), significantly higher than in non-TNBC (1.4%, 3/216) (*P* < 0.001).

Interestingly, we found that the positivity rate of CAV-1 in breast cancer without axillary lymph node metastasis (13.6%, 21/154) was significantly higher than that in patients with axillary lymph node metastasis (4.9%, 7/143) (*P* = 0.010). We collected D2–40 IHC staining data during the postoperative pathological assessment of this cohort of breast cancer cases. Since D2–40 detection was not performed in 144 cases at that time, D2–40 results were available for only 156 cases, among which 29 were diagnosed with lymphatic vessel invasion. Baseline characteristics of the patient groups with or without available D2–40 data are presented in **Table 3**. We then further analyzed the relationship between CAV-1 expression and lymphatic invasion. Consistent with the results of axillary lymph nodes, the positivity rate of CAV-1 in patients negative for lymphatic vessel invasion was 12.6% (16/127), significantly higher than the 0.0% (0/29) in patients positive for lymphatic vessel invasion, but no statistical difference was observed (*P* = 0.093). Furthermore, we analyzed the relationship between CAV-1 expression and axillary lymph node status within the TNBC group. The CAV-1 positivity rate was 34.5% (19/55) in the node-negative group and 25.0% (6/24) in the node-positive group, with no statistically significant difference between the two groups (*P* = 0.402).

**Table 3:** Baseline characteristics of the patient groups with or without available D2–40 data.

Variables	Without D2–40 data, n (%)	With D2–40 data, n (%)	*P*-value
Age (years)			
≤35	5 (3.5%)	9 (5.8%)	0.308
36–55	91 (63.2%)	86 (55.1%)	
>55	48 (33.3%)	61 (39.1%)	
Tumor size (cm)			
≤2	64 (44.4%)	79 (50.6%)	0.283
>2	80 (55.6%)	77 (49.4%)	
Lymph node metastases			
No	66 (46.8%)	88 (56.4%)	0.098
Yes	75 (53.2%)	68 (43.6%)	
Tumor grade			
I	7 (4.9%)	6 (3.8%)	0.055
II	97 (67.4%)	86 (55.1%)	
III	40 (27.8%)	64 (41.0%)	
Tumor stage			
I	34 (23.9%)	53 (34.0%)	0.088
II	71 (50.0%)	75 (48.1%)	
III–IV	37 (26.1%)	28 (17.9%)	
Molecular classification			
non-TNBC	113 (79.0%)	103 (66.5%)	0.015
TNBC	30 (21.0%)	52 (33.5%)	

In multiple logistic regression analysis, the independent predictor of CAV-1 expression in breast cancer was TNBC status (odds ratio = 32.099; 95% confidence interval: 9.341–110.302; *P* < 0.001).

### The value of CAV-1 expression for diagnosing TNBC

**Figure 2:**
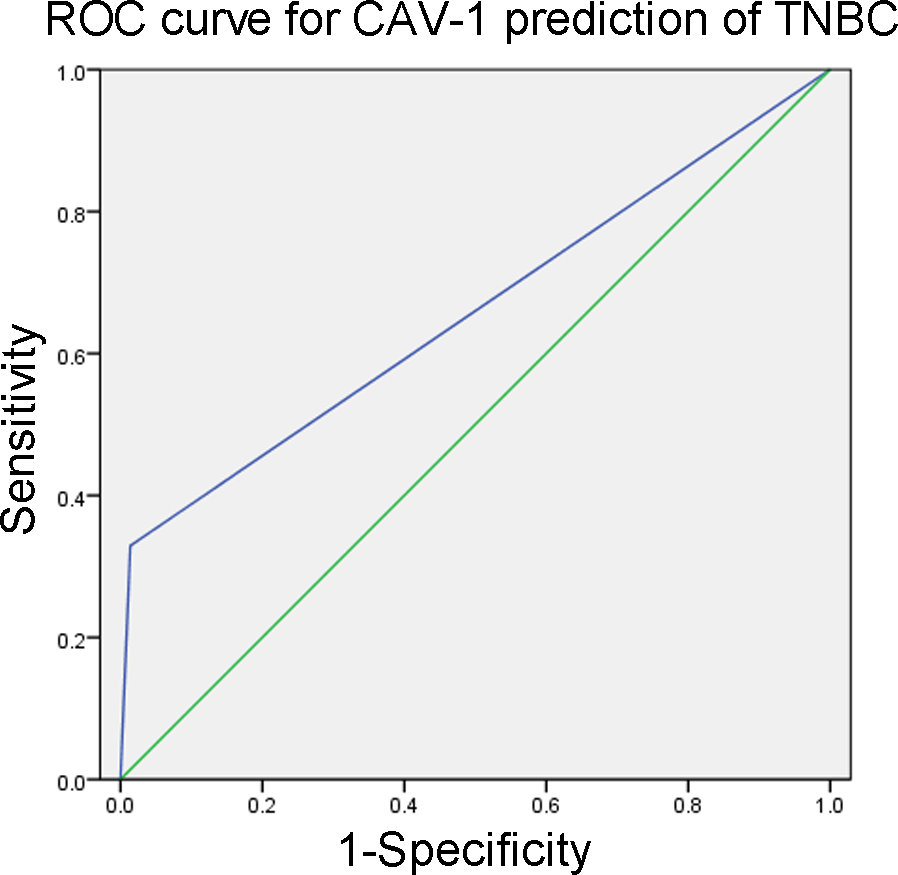
ROC curve for CAV-1 prediction of TNBC.

Given the high expression of CAV-1 in TNBC, we conducted a further analysis of the value of CAV-1 expression in the diagnosis of TNBC. The ROC curve analysis indicated that the area under the curve (AUC) for predicting TNBC with positive CAV-1 was 0.658 (95% *CI* 0.581-0.734; *P* < 0.001; **Figure 2**), yielding a sensitivity of 32.9%, specificity of 98.6%, PPV of 90.0%, and NPV of 79.5%. The corresponding positive likelihood ratio was 23.5 and the negative likelihood ratio was 0.681, indicating that a positive test result is 23.5 times more likely to occur in patients with TNBC than in those without.

### No association between CAV-1 expression and survival

**Figure 3:**
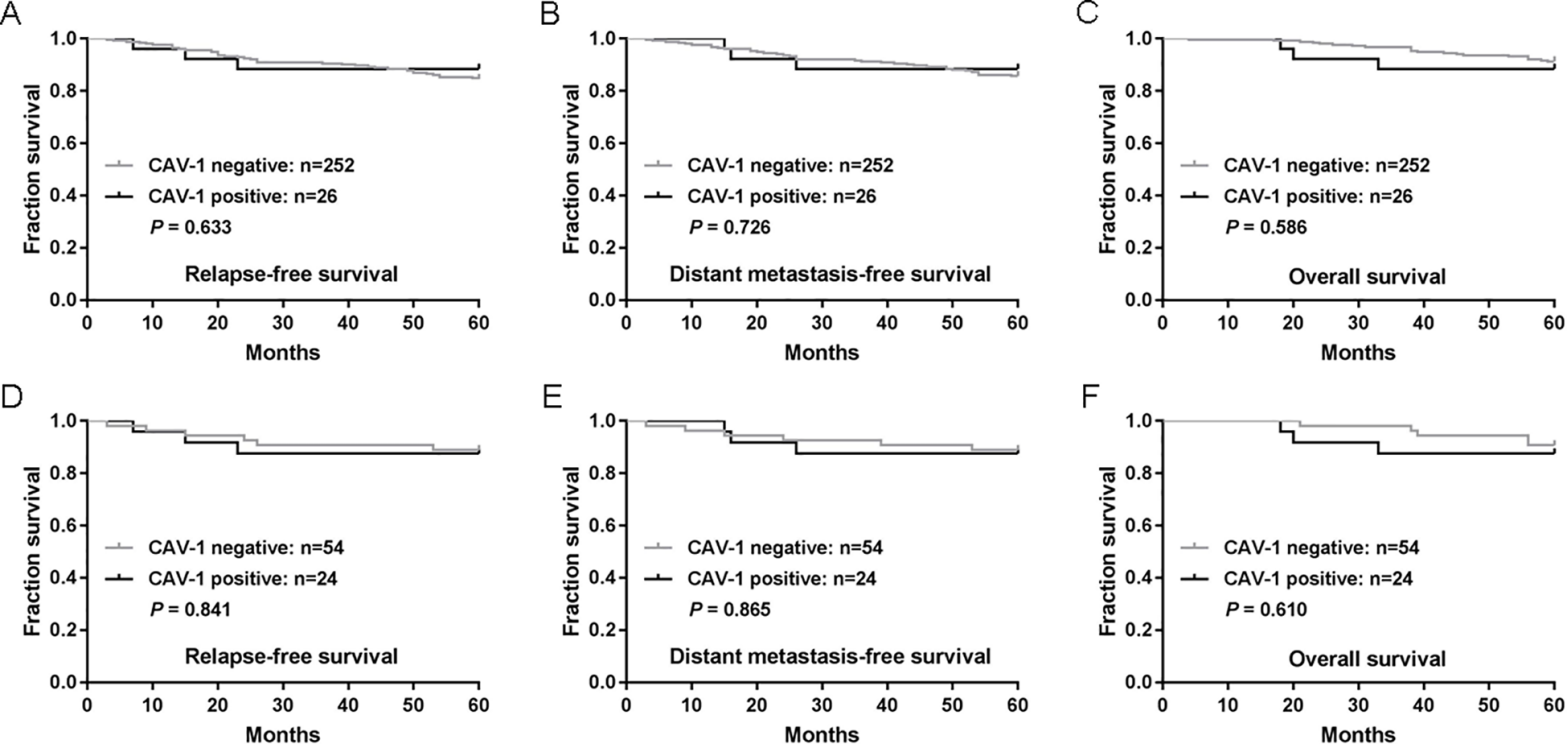
CAV-1 expression is not associated with the survival of patients with breast cancer. **(A–C)** The associations of CAV-1 expression with relapse-free survival (RFS) **(A)**, distant metastasis-free survival (DMFS) **(B)** and overall survival (OS) **(C)**. **(D–F)** The associations of CAV-1 expression with RFS **(D)**, DMFS **(E)** and OS **(F)** of patients with TNBC. *P*-values were calculated using the Mantel-Cox log-rank test.

To assess the potential impact of CAV-1 expression on patient survival, we analyzed CAV-1 expression in relation to RFS, DMFS and OS rates in patients with breast cancer. Five-year RFS, DMFS and OS rates were 84.9%, 86.0% and 91.0%, respectively. As shown in **Figures 3A–C**, no clear associations were observed between CAV-1 expression and these survival variables (*P*>0.05 for each comparison).

We further analyzed the prognostic impact of CAV-1 expression in the TNBC subgroup. The five-year rates of RFS, DMFS, and OS in TNBC patients were 88.5%, 88.5%, and 89.7%, respectively. As shown in **Figures 3D–F**, in TNBC, the prognosis of tumors that were positive CAV-1 expression did not differ significantly from that of negative CAV-1 group (*P*>0.05 for each comparison).

In Cox proportional hazards regression analysis, the results showed that compared to individuals ≤35 years old, patient age between 36–55 years old is an independent protective factor for RFS (hazard ratio [*HR*]=0.249, 95% *CI* = 0.069–0.896, *P* = 0.033), DMFS (0.222, 0.061–0.810, *P* = 0.023) and OS (0.092, 0.021–0.399, *P* = 0.001); On the other hand, stage III–IV tumor is an independent unfavorable prognostic factor for RFS (6.871, 2.540–18.588, *P* < 0.001), DMFS (6.442, 2.363–17.563, *P* < 0.001) and OS (22.099, 2.825–172.844, *P* = 0.003).

## Discussion

CAV-1 is involved in regulating various cellular functions and may be related to breast cancer development (3–9). Limited studies have examined CAV-1 expression in breast cancer tissues using immunohistochemistry (10, 11). Results suggest that high CAV-1 levels may be linked to improved survival in patients who received Neoadjuvant chemotherapy. However, the small number of studies and specific subtypes of breast cancer analyzed have not fully revealed the significance of CAV-1 in breast cancer.

The findings of the present study demonstrate a significant association between CAV-1 expression and multiple clinicopathological characteristics of breast cancer. The positivity rate of CAV-1 was significantly higher in breast cancer tissues compared with normal breast tissues, suggesting a potential involvement of CAV-1 in breast tumorigenesis. Notably, analysis of histological subtypes revealed marked heterogeneity in CAV-1 positivity rates. While invasive ductal carcinoma exhibited a relatively low positivity rate (8.7%), metaplastic carcinoma showed the highest CAV-1 expression (66.7%), followed by medullary carcinoma (20.0%), whereas no CAV-1 positivity was observed in invasive micropapillary carcinoma. These findings suggest that CAV-1 expression may be preferentially associated with specific histological subtypes, particularly metaplastic carcinoma, which is known to be an aggressive and often triple-negative phenotype (21, 22). Furthermore, CAV-1 expression was significantly correlated with aggressive tumor features, including larger tumor size (>2 cm), higher histological grade (grade III), negative hormone receptor status (ER- and PR-), high Ki-67 index (≥14%), and negative HER2 expression (0–1+). These results further substantiate the association between CAV-1 expression and biologically aggressive breast cancer phenotypes. In particular, the positivity rate of CAV-1 in TNBC was markedly higher than in non-TNBC subtypes (32.9% vs. 1.4%). Multivariate logistic regression analysis further identified TNBC status as an independent predictor of CAV-1 expression. TNBC is widely recognized as a breast cancer subtype associated with poor prognosis and limited therapeutic options (23–25). Collectively, our findings suggest that CAV-1 may serve as a potential biomarker for identifying TNBC patients and could offer insights into subtype-specific tumor biology, with possible implications for the development of targeted therapeutic strategies.

Interestingly, we observed a higher positivity rate of CAV-1 in breast cancer patients without axillary lymph node metastasis compared to those with metastasis. Furthermore, our analysis of D2–40 detection data showed that CAV-1 positivity was also higher in patients negative for lymphatic vessel invasion compared to those positive for lymphatic vessel invasion. Studies show that TNBC subtype had lower odds of LVI (26, 27) and axillary lymph node involvement (27–30) relative to other subtypes. Combining the results of this study, TNBC status is an independent related factor of CAV-1 expression. We hypothesized that the apparent protective effect of CAV-1 against lymph node metastasis might be attributable to its strong association with the TNBC phenotype, which itself has a lower propensity for nodal involvement. To further validate this hypothesis, we performed a stratified analysis within the TNBC subgroup, which revealed no significant difference in CAV-1 positivity between node-negative and node-positive patients. This finding provides evidence that the inverse association between CAV-1 expression and lymph node metastasis observed in the overall cohort is driven by the enrichment of CAV-1 positivity in the TNBC subtype. It may be one of the explanations for the low incidence of lymph node metastasis in breast cancer with positive CAV-1 expression.

The results of our study suggest that CAV-1 expression may have potential value in diagnosing TNBC. The ROC curve analysis demonstrated a moderate AUC (0.658), indicating that CAV-1 expression can distinguish TNBC from non-TNBC with reasonable accuracy. Notably, the specificity (98.6%) and PPV (90.0%) of CAV-1 for predicting TNBC were high, suggesting that in clinical practice, a positive CAV-1 result would strongly indicate TNBC. The positive likelihood ratio was 23.5, which indicates that a positive test is over 23 times more likely to occur in patients with TNBC compared to those without, further supporting the utility of CAV-1 as a confirmatory diagnostic marker. However, the sensitivity of CAV-1 was relatively low (32.9%), limiting its ability to serve as an effective standalone screening tool for TNBC. Most TNBC cases in our cohort were CAV-1 negative, as reflected in the NPV of 79.5%. Although the NPV might initially appear modest given the high specificity, it should be interpreted within the context of our study cohort, in which the prevalence of TNBC was 27.3%. In such a setting, markers with high specificity but low prevalence of the target condition produce NPV values that are highly dependent on disease prevalence. To provide a prevalence-independent assessment, we also report the positive and negative likelihood ratios (23.5 and 0.681, respectively). The positive likelihood ratio confirms the strong rule-in potential of CAV-1 expression, whereas the modest negative likelihood ratio further illustrates the limited rule-out value. Consequently, while a positive CAV-1 result is highly suggestive of TNBC, CAV-1 alone may not be sufficient for accurately diagnosing TNBC, especially as a rule-out test. Future studies should explore whether combining CAV-1 with other biomarkers or clinical parameters can improve the diagnostic accuracy for TNBC.

Previous reports have demonstrated an upregulation of CAV-1 in basal-like TNBC cells (31–33), supporting a potential link between CAV-1 expression and basal-like features. Other studies have shown that CAV-1 knockdown enhances radiosensitivity in basal-like TNBC cells by impairing DNA repair via delayed EGFR nuclear translocation and promoting G_2_/M arrest and apoptosis (34). Moreover, studies suggest that CAV-1 may contribute to mesenchymal phenotypes, as its knockdown in breast cancer cells resulted in downregulation of MMPs and upregulation of E-cadherin-indicating a transition toward an epithelial state (35). Thus, CAV-1 upregulation in TNBC may be associated with both basal-like identity and mesenchymal characteristics, highlighting its role in aggressive tumor behavior.

Our survival analysis did not reveal a statistically significant association between CAV-1 expression and survival outcomes. Specifically, no significant differences were observed in the 5-year RFS, DMFS, and OS rates between patients with positive and negative CAV-1 expression. However, given the limited number of CAV-1 positive cases (n=26) in the survival cohort, the study was insufficiently powered to detect clinically meaningful differences. Therefore, the present findings should not be interpreted as definitive evidence against a prognostic role of CAV-1, and the absence of a significant association may reflect inadequate sample size rather than a true lack of effect. Additionally, when we specifically examined the impact of CAV-1 expression on the prognosis of TNBC, we again found no significant differences in survival outcomes between groups. It is important to note that this subgroup analysis, comprising only 24 CAV-1 positive and 54 CAV-1 negative TNBC patients, was particularly underpowered and should be interpreted with caution. Whether expanding the sample size or extending the follow-up duration will clarify the prognostic value of CAV-1 remains to be determined in future studies with adequate statistical power.

The literature reveals a seemingly conflicting duality in CAV-1’s roles, with both tumor-suppressive and oncogenic functions reported (36). Our study observed that CAV-1 expression is associated with breast tumorigenesis and aggressive phenotypes, interpreted within the framework of context-dependent cancer biology. This apparent contradiction can be attributed to several factors: CAV-1 function depends on tissue and cancer type, promoting progression in aggressive subtypes like triple-negative breast cancer (36); its compartmental localization leads to opposing roles in stromal versus epithelial cells (37–39), with our work linking epithelial expression to oncogenic signaling; its effect may be stage-dependent, potentially suppressing early tumors but advancing late-stage disease; and its diverse molecular interactions with pathways involving Src kinase, E-cadherin, STAT5a and matrix metalloproteinases (MMPs) ultimately determine its net outcome (35, 40–42). In summary, CAV-1 is not a binary switch but a multifaceted protein whose biological effect is shaped by cellular context, disease stage, and localization. Our findings support the paradigm that, in aggressive breast cancer, tumor cell-specific CAV-1 expression serves as a marker and potential driver of malignancy.

In conclusion, our study found that CAV-1 expression is significantly upregulated in TNBC, and CAV-1 showed potential clinical value for distinguishing between TNBC and non-TNBC. Despite these findings, we did not observe a clear relationship between CAV-1 expression and patient prognosis in our study cohort. Our findings suggest that CAV-1 may serve as a valuable biomarker for distinguishing TNBC and non-TNBC, providing insights for further research and potential clinical application in breast cancer management. Nonetheless, the investigation of CAV-1 in breast cancer tissues has certain limitations, such as being conducted at a single center with restricted sample size, utilizing a retrospective design that may lead to biases, and offering limited evidence regarding its prognostic significance. Additionally, the use of tissue microarrays with duplicate 1 mm cores, while standard for high-throughput analysis, may introduce sampling bias due to intra-tumoral heterogeneity of CAV-1 expression. Consequently, our observed positivity rate of 10.0% may represent an underestimation, warranting validation through whole-section immunohistochemistry on larger cohorts. Furthermore, in terms of survival analysis, it should be acknowledged that the actual group sizes and event counts in our study were relatively small, resulting in insufficient statistical power to reliably detect a survival difference between groups. As such, the null findings regarding CAV-1’s prognostic effect should not be interpreted as definitive evidence for the absence of a prognostic association. Future studies should prioritize prospective cohort designs, longitudinal survival analyses, and assessments in diverse populations to strengthen the diagnostic and prognostic potential of CAV-1. Addressing these limitations is crucial for effectively incorporating CAV-1 into routine clinical practice for breast cancer diagnosis and prognosis.

## Data Availability

The original contributions presented in the study are included in the article/supplementary material. Further inquiries can be directed to the corresponding author.
